# Multi-omics reveals an association of the gut butyrate-IDO1-tryptophan axis with Yinchenhaotang plus Zexietang-ameliorated NASH in a microbiota-dependent manner

**DOI:** 10.1186/s13020-025-01304-w

**Published:** 2026-01-21

**Authors:** Weimeng Liu, Xiaoyu Sha, Na Zhao, Baoying Liu, Guanlin Yang, Lianqun Jia, Guoyuan Sui

**Affiliations:** 1 Key Laboratory of Ministry of Education for Traditional Chinese Medicine Viscera-State Theory and Applications, Liaoning University of Traditional Chinese Medicine, Shenyang, Liaoning People’s Republic of China; 2 School of Basic Medicine, Liaoning University of Traditional Chinese Medicine, Shenyang, Liaoning People’s Republic of China

**Keywords:** Yinchenhaotang plus Zexietang, Nonalcoholic steatohepatitis, Microbiome, Metabolomics, Transcriptome, Tryptophan metabolism

## Abstract

**Background:**

Nonalcoholic steatohepatitis (NASH) is a complex metabolic disorder with limited effective treatments, and its pathogenesis involves intricate crosstalk between gut microbiota, metabolism, and host signaling. Yinchenhaotang plus Zexietang (YCHZX), a traditional Chinese medicine (TCM) formulation, exhibits therapeutic potential in NASH, but its underlying mechanism—especially its interactions with the gut microbiota and metabolic networks—remains unclear.

**Methods:**

A NASH mouse model was established via a high-fat/high-fructose/high-cholesterol diet. Mice were treated with YCHZX or its individual components (YCH, ZX). Serum biochemistry and liver histopathology were used to evaluate systemic therapeutic effects. Integrated multi-omics analyses (16S rRNA microbiome, serum metabolomics, colon transcriptomics) combined with immunofluorescence, immunohistochemistry, RT‒qPCR and ELISA were employed to explore regulatory networks. Complementarily, the effects of sodium butyrate and indolelactic acid (ILA) were investigated using an LPS-stimulated Caco-2 cell model. Antibiotic-mediated gut microbiota ablation was performed to verify microbiota dependency.

**Results:**

YCHZX outperformed YCH and ZX in improving TC, LDL-C and hepatic pathology. Integrated multi-omics analysis demonstrated that the efficacy of YCHZX was associated with a distinct restructuring of the gut microbiota, specifically enriching butyrate-producing genera such as *Lachnospiraceae_NK4A136_group*. Concomitantly, YCHZX intervention suppressed colonic indoleamine 2,3-dioxygenase 1 (IDO1) and significantly elevated serum levels of ILA, a shift validated in vitro by the direct inhibitory effect of sodium butyrate on IDO1. The elevated ILA was shown to strengthen the gut barrier by upregulating occludin expression in LPS-stimulated Caco-2 cells via an aryl hydrocarbon receptor (AhR)-dependent mechanism. Further, YCHZX activated the AhR, upregulating tight-junction proteins (occludin) to reduce lipopolysaccharide (LPS) translocation, and inhibiting hepatic LPS/TLR4 signaling, TG accumulation, and IL-1β inflammation. All these effects of YCHZX were diminished by antibiotic-induced gut microbiota depletion.

**Conclusion:**

Our findings demonstrate that YCHZX alleviates NASH in a gut microbiota-dependent manner. We propose a mechanism whereby YCHZX enriches butyrate-producing bacteria, which is associated with the suppression of colonic IDO1 and a shift in tryptophan metabolism toward ILA production. The increased ILA, in turn, contributes to the activation of the AhR, thereby restoring gut barrier integrity and mitigating liver inflammation.

**Supplementary Information:**

The online version contains supplementary material available at 10.1186/s13020-025-01304-w.

## Introduction

Nonalcoholic fatty liver disease (NAFLD) has emerged as a leading global public health challenge, with a worldwide prevalence estimated at approximately 30.2% [1]. Beyond its well-documented associations with obesity, dyslipidemia, and metabolic syndrome, NAFLD significantly elevates the risk of type 2 diabetes, cardiovascular diseases, and a spectrum of liver-related complications—including advanced fibrosis and hepatocellular carcinoma (HCC) [2–4]. Pathologically, NAFLD encompasses a progressive continuum of hepatic lesions: it initiates as simple steatosis (nonalcoholic fatty liver, NAFL) and progresses to nonalcoholic steatohepatitis (NASH)—a critical intermediate stage affecting about 25% of NAFLD patients [5]. NASH is not only a key driver of NAFLD escalation but also increases the risk of progression to liver fibrosis, cirrhosis, and ultimately HCC [6, 7]. The multifactorial pathogenesis of NASH, which integrates genetic predispositions, environmental triggers (e.g., dietary patterns), and systemic metabolic dysregulation, poses substantial barriers to the development of effective therapeutic interventions—highlighting an urgent need for novel, targeted strategies that address its complex regulatory networks.

A growing body of evidence identifies gut microbiota dysbiosis as a pivotal contributor to NASH pathogenesis, primarily via the induction of gut barrier dysfunction and subsequent dysregulation of the gut-liver axis [8]. The small-molecule metabolites produced by gastrointestinal bacteria serve as critical messengers that mediate crosstalk between the gut microbiota and the host [8–10]. Clinical and preclinical observations consistently link altered levels of key microbial metabolites—including bile acids, short-chain fatty acids (SCFAs), branched-chain amino acids, and tryptophan derivatives—to NASH progression [8–10]. These metabolites modulate immune homeostasis, inflammatory responses, and metabolic balance, while also maintaining gut epithelial integrity; collectively, they influence NASH development and progression by regulating bidirectional communication between the gut and liver [8–10]. Among these metabolites, tryptophan-derived indole derivatives have garnered particular attention: they act as endogenous ligands for the aryl hydrocarbon receptor (AhR), a transcription factor that modulates immune responses, protects gut barrier function, reduces endotoxaemia and chronic inflammation, and regulates hepatic lipid metabolism [11]. Despite this progress, the precise mechanisms by which gut microbiota orchestrate host metabolic pathways (e.g., tryptophan metabolism) to influence NASH development remain incompletely elucidated.

Correcting gut microbiota dysbiosis and restoring dysregulated microbial metabolite profiles using traditional Chinese medicine (TCM)—a therapeutic approach characterized by multi-component and multi-targeted actions—has emerged as a promising strategy for NASH treatment. Intermediate stage of NAFLD often progresses to NASH, predominantly manifesting as dampness-heat syndrome [12, 13]. Modified Yinchenhao Decoction (YCH), formulated by adding *Alisma orientale*, *Atractylodes macrocephala Koidz*, and other herbs to the base formula, is recommended for NASH treatment in multiple consensus guidelines [14, 15]. Historically, the core formula YCH comprise *Artemisia capillaris Thunb*, *Gardenia jasminoides J.Ellis*, and *Rheum palmatum *L [16]. Its action to clear hepatic damp-heat is further recorded in Li Shizhen's Ben Cao Gang Mu [17]. Zexie Decoction (ZX) contains *Alisma orientale* (dampness-draining) and *Atractylodes macrocephala Koidz* (spleen-strengthening) to address water-dampness accumulation and spleen deficiency [18]. Thus, YCH plus ZX (YCHZX) may simultaneously clear heat-dampness and strengthen the spleen, addressing the core pathogenesis of NASH. Evidences showed that YCHZX, YCH, and ZX all ameliorate NASH-related symptoms, with YCHZX demonstrating superior efficacy compared to its individual components [19–21]. Additionally, YCH and ZX have been reported to modulate gut microbiota composition [22–25], suggesting that gut microbiota may be a key mediator of YCHZX’s therapeutic effects. However, critical gaps remain: the mechanism by which YCHZX remodels the gut microbiota, coordinates changes in microbial metabolites, and integrates these effects to alleviate NASH has not been systematically characterized. Specifically, it remains unknown whether YCHZX’s microbiota-modulating effects are linked to key metabolic pathways (e.g., tryptophan metabolism) or host transcriptional programs (e.g., genes regulating gut barrier function) that drive NASH pathogenesis.

To fill these gaps, we conducted a comprehensive, integrated multi-omics study (16S rRNA microbiome, serum metabolomics, colon transcriptomics) in a high-fat/high-fructose/high-cholesterol (HFHFC)-induced NASH mouse model. We further validated key findings using immunofluorescence, immunohistochemistry, and enzyme-linked immunosorbent assay (ELISA), and employed antibiotic-mediated gut microbiota ablation to verify the microbiota dependency of YCHZX’s effects. Our goal was to systematically dissect the regulatory networks underlying YCHZX’s anti-NASH efficacy, with a specific focus on uncovering potential crosstalk between gut microbiota, microbial metabolites, and host genes.

## Methods and materials

### Drugs and reagents

The following drugs and reagents were used in this study: HFHFC diet (D18402002, Changzhou Mouse One Mouse Two Biotechnology Co., Ltd.), with the nutritional composition specified as 40 kcal% fat, 22% fructose, 10% sucrose, 2% cholesterol to align with the diet-induced NASH model’s clinical relevance [26]; neutral paraformaldehyde fixative (4%, G1101, Servicebio); oil red O solution (G1015, Servicebio); haematoxylin solution (G1004, Servicebio); Hematoxylin and Eosin (H&E) staining kit (G1076, Servicebio); methanol (HPLC grade, A456-4, Thermo Fisher Scientific); acetonitrile (HPLC grade, A955-4, Thermo Fisher Scientific); formic acid (LC–MS grade, A117-50, Thermo Fisher Scientific); Butyric acid(Dr. Ehrenstorfer); E.Z.N.A.^®^Stool DNA Kit (Omega Bio-tek); NEB Next Ultra II DNA Library Prep Kit (New England Biolabs); Agilent DNA 1000 Kit (Agilent Technologies); KAPA Library Quantification Kit (KAPA Biosystems); NextSeq 1000/2000 P1 Reagent Kit (600 cycles, PE300, Illumina); NEBNext^®^ Ultra™ RNA Library Prep Kit for Illumina^®^ (NEB); anti-indoleamine 2,3-dioxygenase 1 (IDO1) antibody (1:250 dilution, 13268-1-AP, Proteintech); anti-claudin antibody (1:400 dilution, GB111401-100, Servicebio); anti-occludin antibody (1:400 dilution, GB15149-50, Servicebio); Kynurenine ELISA Kit (ml036253H, Shanghai Enzyme-linked Biotechnology Co., Ltd.); Indole-3-lactic acid (ILA) (MB-66170A, Jiangsu Enzyme Labeling Biotechnology Co., Ltd); Aryl Hydrocarbon Receptor (AhR) ELISA Kit (YJ037807H, Shanghai Yuanju Biological Technology Co., Ltd.); Interleukin-22 (IL-22) ELISA Kit (YJ063138H, Shanghai Yuanju Biological Technology Co., Ltd.); lipopolysaccharide (LPS) ELISA Kit (ml063892H, Shanghai Enzyme-linked Biotechnology Co., Ltd.); Triglyceride (TG) Assay Kit (A110-1-1, Nanjing Jiancheng Bioengineering Institute); Interleukin-1β (IL-1β) ELISA Kit (YJ301814H, Shanghai Yuanju Biological Technology Co., Ltd.);Sodium butyrate (HY-B0350A, MCE); ILA (GC33659, Glipbo); CH-223191(HY-12684, MCE); LPS(GC60995,Glipbo).

### Preparation of YCHZX, YCH and ZX

The composition of YCH is *Artemisia capillaris Thunb* (18 g), *Gardenia jasminoides J.Ellis* (9 g), and *Rheum palmatum L* (6 g). The composition of ZX is *Alisma orientale* (15 g) and *Atractylodes macrocephala Koidz* (6 g). The composition of YCHZX is *Artemisia capillaris Thunb* (18 g), *Gardenia jasminoides J.Ellis *(9 g), *Rheum palmatum L* (6 g), *Alisma orientale* (15 g), and *Atractylodes macrocephala Koidz* (6 g). Equivalent animal doses were calculated from human clinical doses (33 g/day for YCH, 21 g/day for ZX, and 54 g/day for YCHZX) based on body surface area conversion [27]. Herbal materials were soaked in ten volumes of distilled water for 30 min, boiled for 2 h, and filtered. The residue was boiled again with eight volumes of water for 1.5 h. The combined extracts were concentrated and reconstituted with distilled water to final concentrations of 0.495 g/mL (YCH), 0.315 g/mL (ZX), and 0.81 g/mL (YCHZX).

### Characterization and quality control of YCHZX via ultrahigh-performance liquid chromatography‒Q-Orbitrap high-resolution mass spectrometry (UHPLC-Q-Orbitrap HRMS)

YCHZX (1.0 g) was extracted with 40 mL of 80% methanol under ultrasonication for 30 min. After centrifugation (4 °C, 12,000 r/min, 10 min), the supernatant was analyzed using a Vanquish Flex UHPLC system equipped with an ACQUITY UPLC HSS T3 column (2.1 × 100 mm, 1.7 μm). The mobile phase consisted of (A) 0.1% formic acid in water and (B) acetonitrile, with a flow rate of 0.3 mL/min and column temperature of 40 °C. MS analysis was performed in full-scan/dd-MS^2^ mode using a HESI-II probe. Data were processed with Progenesis QI 3.0, and compounds were identified by matching retention times, mass accuracy, fragment patterns, and isotope distributions against a custom database (TCM Pro 2.0, Beijing Hexin Technology Co., Ltd.).

### Animal handling

This study consists of two animal experiments. In the first animal experiment, fifty male C57BL/6 J mice (SPF-grade, aged 6–8 weeks, weighing 18–22 g) were purchased from Liaoning Changsheng Biotechnology Co., Ltd. with licence number SYXK (Liaoning) 2019–0004. They were housed in the Experimental Animal Center of Liaoning University of Traditional Chinese Medicine. The mice were subjected to a 2-week adaptive feeding period and then randomly divided into five groups, i.e., the control, model, YCH, ZX, and YCHZX groups, with 10 mice in each group. Mice in the control group were fed a chow diet, whereas mice in the other groups were fed a HFHFC diet [26]. The mice had free access to food and water. After 20 weeks of continuous feeding, the mice in the YCH, ZX, and YCHZX groups were gavaged daily with 0.3 mL of the YCH, ZX, or YCHZX solutions, respectively. The mice in the control and model groups were gavaged daily with 0.3 mL of saline. This gavage procedure was continued for 8 weeks. In the second animal experiment, antibiotics (Abx) were used to deplete the gut microbiota. A pseudogerm-free (PGF) mouse model was generated using an antibiotic cocktail (neomycin, ampicillin, metronidazole, and vancomycin) to deplete the gut microbiota [28]. Thirty male C57BL/6 J mice were randomly divided into five groups: the control, model, YCHZX, model + abx, and YCHZX + abx groups (n = 6 in each group). Mice in the control group were fed a basic diet, whereas mice in the other groups were fed a HFHFC diet for 14 weeks to induce early NASH features (such as steatosis and inflammation, validated by our preliminary 14-week pilot experiment showing initial pathology (supplementary Fig. 1), and consistent with literature on similar models [29]), followed by 8 weeks of treatment (total 22 weeks). The mice in the YCHZX and YCHZX + abx groups were gavaged daily with 0.3 mL of YCHZX for 8 weeks. The mice in the control and model groups were gavaged daily with 0.3 mL of saline for 8 weeks. Antibiotic treatment was applied for 7 days before YCHZX treatment and was maintained until the end of the experiment. The experimental workflow is schematically outlined in Fig. 2A and Fig. 4A. The experiment was conducted with the approval of the Experimental Animal Ethics Committee of Liaoning University of Traditional Chinese Medicine with ethics review numbers 2021000042022098.

### Cell culture and treatments

Caco-2 cells (Servicebio, STCC108108P) were cultured in MEM containing 20% fetal bovine serum (FBS) and 1% penicillin–streptomycin at 37 °C in a 5% CO₂ atmosphere. For cell viability assessment, cells were seeded into plates and exposed to ILA or CH-223191 for 24 h and 48 h, after which viability was evaluated using the CCK-8 assay. The experimental groupings were as follows: Experiment 1 (24 h): control, LPS (10 μg/mL) [30], LPS + butyrate (0.5 mmol/L) [31]; Experiment 2 (48 h): control, LPS, LPS + ILA (0.5 mmol/L) [30, 32], LPS + CH-223191 (10 μmol/L) + ILA [30, 33].

### Histopathological examination

Paraformaldehyde-fixed liver tissues were dehydrated through a graded ethanol series (70%, 80%, 90%, 95%, and 100%), cleared in xylene, and embedded in paraffin. Paraffin-embedded liver Sects. (4 μm) were stained with H&E using a standard kit: deparaffinized in xylene (3 × 5 min), rehydrated through graded ethanol (100%, 95%, 90%, 80%, 70%, 5 min each), stained with haematoxylin for 5 min, differentiated in 1% hydrochloric acid–ethanol for 30 s, blued in 1% ammonia water for 1 min, stained with eosin for 3 min, dehydrated, cleared, and mounted with neutral balsam. All stained sections were imaged using a light microscope at 200 × magnification. Liver NAFLD Activity Score (NAS) was evaluated by two independent pathologists who were blinded to the experimental groups, following the NASH Clinical Research Network (CRN) criteria [34]: steatosis (0–3 points, hepatocellular ballooning (0–2 points), and lobular inflammation (0–3 points). The total NAS was the sum of the three components (range: 0–8). Colon tissues were processed similarly to liver tissues for H&E staining (4 μm paraffin sections).

### Fully automatic biochemical analysis

A fully automatic biochemical analyser was used to measure the serum TC, TG, low-density lipoprotein cholesterol (LDL-C), high-density lipoprotein cholesterol (HDL-C), aspartate aminotransferase (AST) and alanine aminotransferase (ALT) levels.

### Amplification of the V3-V4 region of the 16S rRNA gene in the caecal contents

Genomic DNA was extracted from cecal content samples using the OMEGA Soil DNA Kit (Omega Bio-Tek, USA). The quality and concentration of the extracted DNA were assessed with a Nanodrop 2000 spectrophotometer (Thermo Fisher Scientific, USA). The V3–V4 hypervariable region of the bacterial 16S rRNA gene was amplified by PCR. The resulting amplicons were purified, and sequencing libraries were constructed following standard protocols. Library quality control included fragment size analysis on an Agilent 2100 bioanalyzer (Agilent Technologies, USA) and quantification using an ABI StepOnePlus Real-Time PCR System (Applied Biosystems, USA). Finally, paired-end sequencing (2 × 300 bp) was performed on the Illumina MiSeq platform (Illumina, USA).

Raw sequencing reads were processed as follows. Quality control and adapter trimming were conducted using fastp (v0.20.0) with the following parameters: reads were trimmed from the 3ʹ-end until a base quality ≥ 20 was reached; a 50-bp sliding window was applied, and the trailing portion was truncated if the average quality within the window fell below 20; reads shorter than 50 bp or containing ambiguous nucleotides ("N") were discarded. Paired-end reads were then merged using FLASH (v1.2.7), with a minimum overlap of 10 bp and a maximum mismatch ratio of 0.2. Demultiplexing and orientation of sequences were performed based on barcode and primer sequences, allowing zero mismatches in barcodes and up to two mismatches in primers. Subsequent bioinformatic analysis was performed within the QIIME 2 framework (v2020.2). Quality-filtered sequences were denoised, and amplicon sequence variants (ASVs) were inferred using the DADA2 plugin with default parameters, which also removed chimeric sequences. The resulting ASV table was rarefied to an even sequencing depth across all samples to minimize bias in downstream diversity analyses. Taxonomic assignment of representative ASVs was carried out using a naive Bayes classifier trained on the SILVA 138 database. Alpha and beta diversity analyses were conducted based on the rarefied ASV table. Beta diversity was measured using weighted and unweighted UniFrac distances, and results were visualized via principal coordinates analysis (PCoA). Taxonomic composition was summarized and displayed in bar plots at the genus level. Differential abundance of taxa between groups was assessed using the Kruskal–Wallis test. Functional potential of the microbial communities was predicted with PICRUSt2, and differentially enriched metabolic pathways were identified using linear discriminant analysis effect size (LEfSe), with an LDA score threshold of > 2.0 considered significant.

### Untargeted serum metabolomics

Fifty microlitres of each serum sample was mixed with 300 μL of internal standard extraction solution (20% acetonitrile-methanol), vortexed for 3 min, and centrifuged at 12,000 r/min for 10 min at 4 °C. After centrifugation, 200 μL of the supernatant was transferred to a new centrifuge tube, which was left to stand at -20 °C for 30 min and then centrifuged again at 12,000 r/min for 3 min at 4 °C. Subsequently, 180 μL of the supernatant was transferred for analysis. The chromatographic column used was a Waters ACQUITY Premier HSS T3 (1.8 µm, 2.1 mm × 100 mm). Mobile phase A was 0.1% formic acid in water, and mobile phase B was 0.1% formic acid in acetonitrile. The column temperature was set at 40 °C, the flow rate was 0.4 mL/min, and the injection volume was 4 μL. The raw data were converted to mzXML format using ProteoWizard and subjected to peak extraction, alignment, and retention time correction using the XCMS program. The corrected and filtered peaks were identified by searching a self-built database that included data from public and predictive databases via the metDNA method. Data analysis included qualitative and quantitative analyses of the metabolites, sample quality control analysis, orthogonal partial least squares discriminant analysis (OPLS-DA), screening of differentially abundant metabolites (*P* < 0.05 and VIP > 1 as the threshold for significant differential metabolites), and KEGG functional annotation and enrichment analysis of the differentially abundant metabolites.

### Colon transcriptomic analysis

Total RNA was extracted from the samples via the TRIzol method. After the RNA samples were qualified, 1.5 μg of RNA from each sample was used for library preparation and sequencing. The raw data (raw reads) obtained from sequencing were first filtered using in-house Perl scripts and then aligned to the reference genome using HISAT2 software (v2.1.0). The gene expression levels in each sample were analysed using featureCounts software (v1.5.0p3). Differential gene expression analysis was conducted on the basis of the read count data obtained from gene expression level analysis. DESeq2 was used for differential expression analysis with the general criterion of a *P* value < 0.05 and |logFC|> 1.

### GC/MS

The cecal contents was accurately weighed into a 1.5 mL EP tube. Then, 0.05 mL of 50% H_2_SO_4_ and 0.2 mL of an internal standard extraction solution (25 mg/L in methyl tert-butyl ether) were added sequentially. The mixture was vortexed for 30 s and mechanically oscillated for 10 min, followed by ultrasound extraction for 10 min in an ice-water bath. The extract was subsequently centrifuged at 10,000 rpm and 4 °C for 15 min. All samples were then maintained at −20 °C for 30 min. Finally, the upper organic supernatant was carefully transferred into a fresh 2 mL glass vial for GC–MS analysis. Analysis was performed using an Agilent 7890B-5977B GC–MS system equipped with an HP-FFAP capillary column. A 1 µL aliquot was injected in split mode with a 5:1 ratio. High-purity helium was used as the carrier gas at a constant column flow rate of 1.2 mL/min, with a front inlet purge flow of 3 mL/min. The GC oven temperature program was initialized at 50 °C for 1 min, then ramped to 150 °C at 50 °C/min and held for 1 min, further increased to 170 °C at 10 °C/min, then raised to 225 °C at 25 °C/min and held for 1 min, and finally elevated to 240 °C at 40 °C/min for a final 1-min hold. The temperatures for the injection port, transfer line, ion source, and quadrupole were set at 220 °C, 240 °C, 240 °C, and 150 °C, respectively. Electron impact ionization was employed with an electron energy of −70 eV.

### Immunofluorescence

IDO1 expression was evaluated via immunofluorescence. Paraffin-embedded sections were deparaffinized in water, antigen retrieval was performed, and the sections were dried slightly by shaking. Then, a circle was drawn around the tissue using a tissue marker, and bovine serum albumin (BSA) was added dropwise to block nonspecific binding for 30 min. The prepared primary antibody (IDO1) was added dropwise, and the sections were incubated overnight at 4 °C in a wet box. The glass slide was then washed three times with phosphate-buffered saline (PBS). The corresponding secondary antibody was added, and the samples were incubated at room temperature in the dark for 50 min. DAPI was used to counterstain the cell nuclei, the spontaneous fluorescence of the tissue was quenched, and an anti-fluorescence quenching sealing agent was applied before image acquisition.

### Immunohistochemistry

Claudin and occludin expression was evaluated via immunohistochemistry. Paraffin-embedded sections were deparaffinized in water, antigen retrieval was performed, endogenous peroxidase activity was blocked, serum blocking was performed, and the blocking solution was removed with gentle shaking. Primary antibodies were added dropwise to the sections, which were incubated overnight at 4 °C in a wet box. The glass slides were washed with PBS, dried slightly, and incubated with the secondary HRP-labelled antibody at room temperature for 50 min. Then, the glass slides were washed three times with PBS and dried slightly, after which freshly prepared DAB solution was added dropwise. The colour development time was controlled by microscopic observations. The result was considered positive if a brownish yellow colour appeared, after which the slides were rinsed with tap water to terminate colour development. The cell nuclei were stained, and the samples were dehydrated, sealed, and placed under a light microscope for analysis.

### ELISA and GPO-PAP

The concentrations of kynurenine, AhR, and IL-22 in colon tissue; ILA in serum; LPS in serum and cecal contents; and IL-1β in liver tissue were measured using commercial ELISA kits according to the manufacturers’ instructions. Liver TG levels were quantified using the GPO-PAP method as per the manufacturer’s protocol.

### Reverse transcription quantitative polymerase chain reaction (RT-qPCR)

The mRNA level of Toll-like receptor 4 (TLR4), Cytochrome P450 Family 1 Subfamily A Member 1(Cyp1A1), and occludin was tested by RT-qPCR. After the liver tissue (0.05 g) was weighed, total RNA was extracted from the liver tissue using an animal total RNA extraction kit. Total RNA in Caco-2 cells was extracted via TRIzol extraction. The reaction system was prepared at 4 °C according to the RNA concentration and to the instructions. RT-qPCR was performed using SYBR Green, and the results were analysed for relative quantification using the 2^−△△Ct^ method. The nucleotide sequences of the target genes were retrieved from the NCBI Nucleotide database. Specific primer pairs were designed using Primer Premier 5.0 software. All primers were synthesized by Servicebio Co., Ltd. (Wuhan, China) with standard desalting purification. The detailed information on the gene primers utilized in the present study can be found in Table 1, and Hypoxanthine phosphoribosyl transferase 1 (Hprt1) was employed as a reference gene for normalization purposes.

**Table 1:** The primer sequences

Gene	Forwards primer	Reverse primer	bp
TLR4	F:TGAGGACTGGGTGAGAAATGAGC	R:CTGCCATGTTTGAGCAATCTCAT	223
Cyp1A1	TCATCCCTATTCTTCGCTACCTAC	CCAAAGAGGTCCAAGACGATGT	122
Occludin	AAGAGTACATGGCTGCTGCTGA	CTTCTTGATGTGTGACAATTTGCTC	241
Hprt1	F:TCATGGACTGATTATGGACAGGACT	R:GCTTTAATGTAATCCAGCAGGTCAG	138

### Western blot

Caco-2 cell lysates were prepared using RIPA buffer supplemented with protease and phosphatase inhibitors. Following centrifugation, the protein concentration of the supernatant was determined by a BCA assay. Equal amounts of proteins were separated by SDS-PAGE and transferred to PVDF membranes. The membranes were blocked with 5% non-fat milk and then incubated with primary antibodies overnight at 4 °C, followed by incubation with HRP-conjugated secondary antibodies for 1 h at room temperature. After extensive TBST washes, immunoreactive bands were visualized using an ECL substrate.

### Statistical analysis

Student’s t test or the Mann‒Whitney U test was used for statistical comparisons between two groups. The Kruskal‒Wallis test or ANOVA was used for analyses involving multiple groups. All the statistical analyses were performed using GraphPad Prism 9 or R Software. Statistical significance was defined as a *P* value < 0.05.

## Results

### Chemical components of YCHZX

**Fig. 1:**
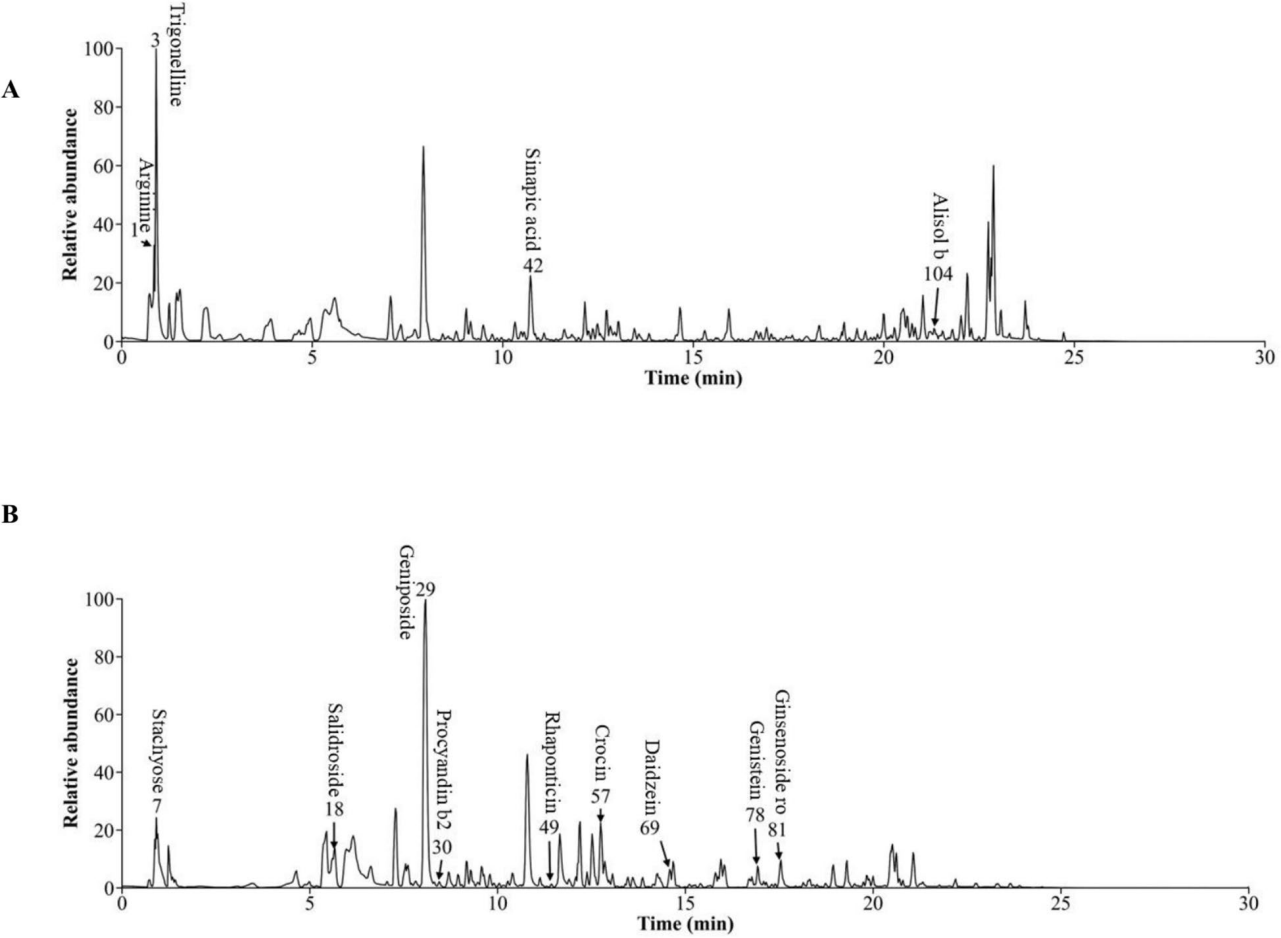
Chemical characterization of YCHZX. **A** Positive ion mode; **B** Negative ion mode. YCHZX: Yinchenhao Tang plus Zexie Tang

UHPLC-Q-Orbitrap HRMS was performed for YCHZX quality control by identifying the ingredients. A total of 110 components were identified, including Arginine, Trigonelline, Stachyose, Salidroside, Geniposide, Procyandin b2, Sinapic acid, Rhaponticin, Crocin, Daidzein, Genistein, Ginsenoside ro, Alisol b, and so on. The positive and negative ion chromatographs for YCHZX are shown in Fig. 1, and the 110 compounds and their basic formulae and structures are displayed in Supplementary Table 1.

### YCHZX improved the pathological features, elevated serum lipid levels and elevated liver enzyme levels in NASH model mice

**Fig. 2:**
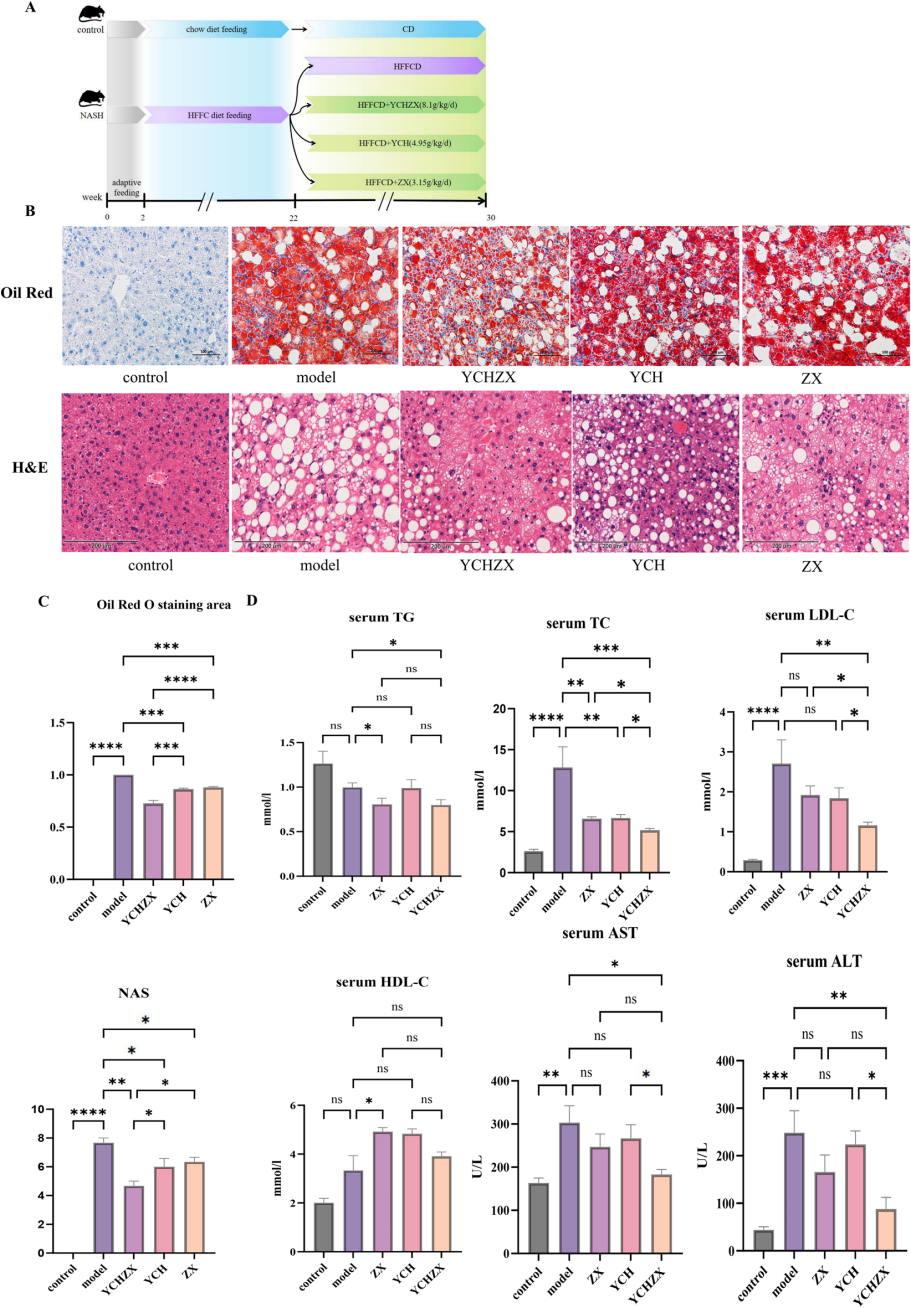
YCHZX ameliorates pathological features, serum lipid profiles, and liver enzyme levels in NASH model mice. **A** Schematic of the animal experimental timeline in NASH model mice; **B** Oil Red O and H&E staining of liver Sects. (200 ×); **C** Oil Red O and H&E staining figures with quantitative data (n = 3); **D** Serum lipid levels and liver enzyme activities (n = 7). Data are presented as mean ± SE. *P < 0.05, **P < 0.01, ***P < 0.001, ****P < 0.0001. YCHZX: Yinchenhao Tang plus Zexie Tang; YCH: Yinchenhao Tang; ZX: Zexie Tang; TG: triglyceride; TC: total cholesterol; HDL-C: high-density lipoprotein cholesterol; LDL-C: low-density lipoprotein cholesterol; AST: aspartate aminotransferase; ALT: alanine aminotransferase; NASH: nonalcoholic steatohepatitis

As shown in Fig. 2B and Fig. 2C, hepatocytes in the control group showed no evident lipid droplets. In contrast, the model group exhibited diffuse fatty degeneration, marked by circular vacuoles of varying sizes along with inflammatory cell infiltration. Compared with the model group, all treatment groups (YCH, ZX, and YCHZX) showed varying degrees of improvement in hepatic steatosis, vacuolation, and inflammatory infiltration, with the most pronounced effect observed in the YCHZX group. As illustrated in Fig. 2D, serum levels of TC, LDL-C, ALT, and AST were significantly elevated in the model group compared to the control group. Conversely, in contrast, YCHZX treatment significantly reduced the levels of TC, TG, LDL-C, ALT, and AST relative to the model group.YCH and ZX treatment significantly reduced the levels of TC relative to the model group. The YCHZX group exhibited the most substantial reductions in both TC and LDL-C levels.

### YCHZX alleviated gut dysbiosis in NASH model mice

**Fig. 3:**
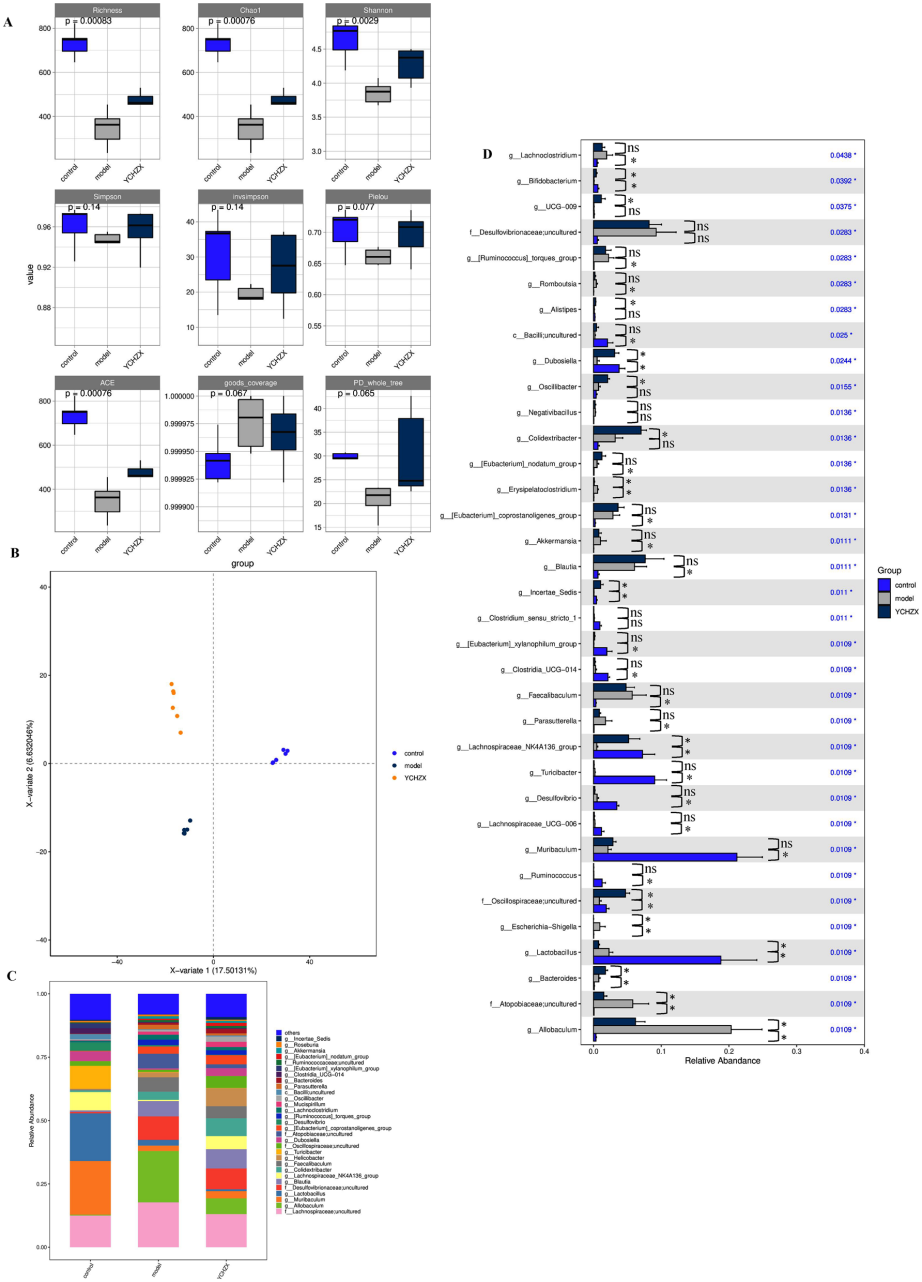
YCHZX alleviates gut microbiota dysbiosis in NASH model mice. **A** α-diversity indices; **B** PLS-DA result; **C** Bacterial taxonomic composition at the genus level (top 30 genera); **D** Comparison of genus-level composition across groups (n = 6). *P < 0.05, **P < 0.01, ***P < 0.001. YCHZX: Yinchenhao Tang plus Zexie Tang; NASH: nonalcoholic steatohepatitis

The gut microbiota plays a critical role in the pathogenesis of NASH, and modulating its composition represents a promising therapeutic strategy. To assess the impact of YCHZX on the gut microbiota, we performed 16S rRNA gene sequencing targeting the V3–V4 region using cecal content samples. Analysis of α-diversity (Chao1, richness, shannon, ACE) revealed significant differences among the control, model, and YCHZX groups (Fig. 3A). In contrast, PLS-DA clearly separated the microbial communities across groups (Fig. 3B), indicating distinct compositional shifts. Taxonomic profiling at the genus level identified the 30 most abundant genera (Fig. 3C). Compared with the control group, the model group exhibited a significant increase in the relative abundance of *Atopobiaceae;uncultured*, *Allobaculum*, *Erysipelatoclostridium*, and *Escherichia-Shigella*. Conversely, notable decreases were observed in *Lachnospiraceae_NK4A136_group*, *Incertae_Sedis, Dubosiella*, *Oscillospiraceae;uncultured*, and *Bifidobacterium* (Fig. 3D). YCHZX treatment restored the abundance of these bacteria compared to the model group. Interestingly, although no significant differences were observed between the model and control groups for *Colidextribacter* and *Oscillibacter*, YCHZX significantly enhanced their relative abundances (Fig. 3D).

### YCHZX attenuated NASH in a gut microbiota-dependent manner

**Fig. 4:**
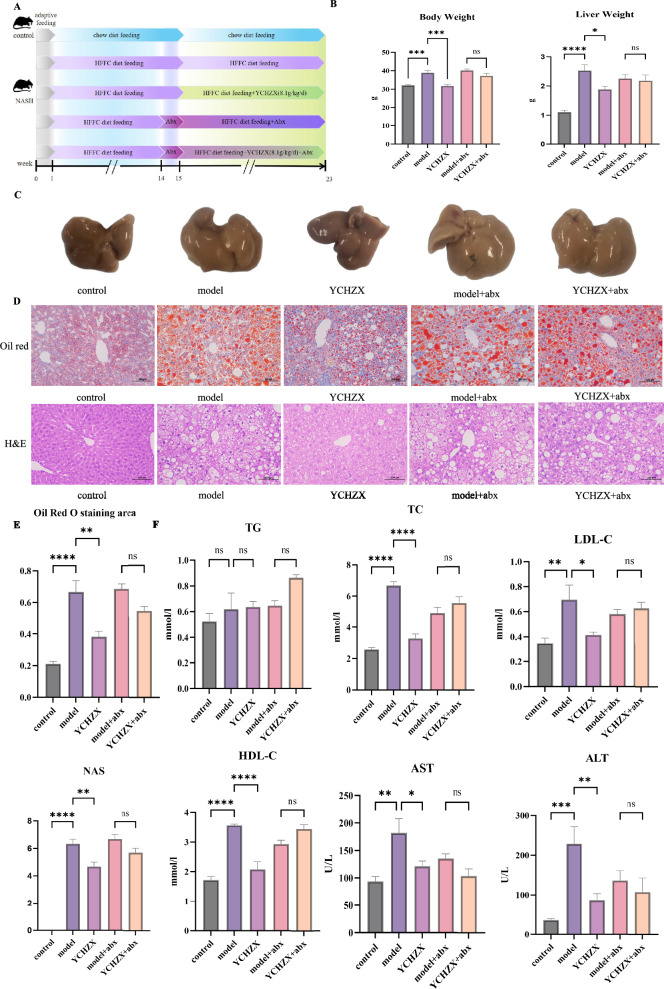
YCHZX attenuates NASH in a gut microbiota-dependent manner. **A** Schematic of the animal experimental timeline in PGF mice; **B** Body weight and liver weight (n = 5); **C** Macroscopic liver morphology; **D** Oil Red O and H&E staining of liver Sects. (200 ×); **E** Oil Red O and H&E staining figures with quantitative data (n = 3); **F** Serum lipid levels and liver enzyme activities (n = 5). Data are presented as mean ± SE. *P < 0.05, **P < 0.01, ***P < 0.001, ****P < 0.0001. YCHZX: Yinchenhao Tang plus Zexie Tang; TG: triglyceride; TC: total cholesterol; HDL-C: high-density lipoprotein cholesterol; LDL-C: low-density lipoprotein cholesterol; AST: aspartate aminotransferase; ALT: alanine aminotransferase; NASH: nonalcoholic steatohepatitis; PGF: pseudogerm-free

To determine whether the therapeutic effects of YCHZX depend on gut microbiota modulation, we administered a broad-spectrum antibiotic cocktail (vancomycin, neomycin sulfate, metronidazole, and ampicillin) to both NASH model mice and YCHZX-treated NASH mice. Compared with the control group, mice in the model group exhibited significant increases in body and liver weights. YCHZX treatment reduced these increases; however, this beneficial effect was diminished following antibiotic-mediated gut microbiota depletion (Fig. 4B). Morphological assessment of liver tissues showed a pronounced yellow discoloration in the model group, consistent with substantial lipid accumulation. YCHZX intervention markedly attenuated this discoloration, but again, the improvement was diminished upon antibiotic-induced microbiota ablation (Fig. 4C). Consistent with these findings, YCHZX ameliorated histopathological abnormalities and reduced elevated serum lipid levels in NASH mice—effects that were also diminished by antibiotic treatment (Fig. 4D-F). These results demonstrate that the anti-NASH effects of YCHZX are gut microbiota-dependent.

### YCHZX targeted tryptophan metabolism

**Fig. 5:**
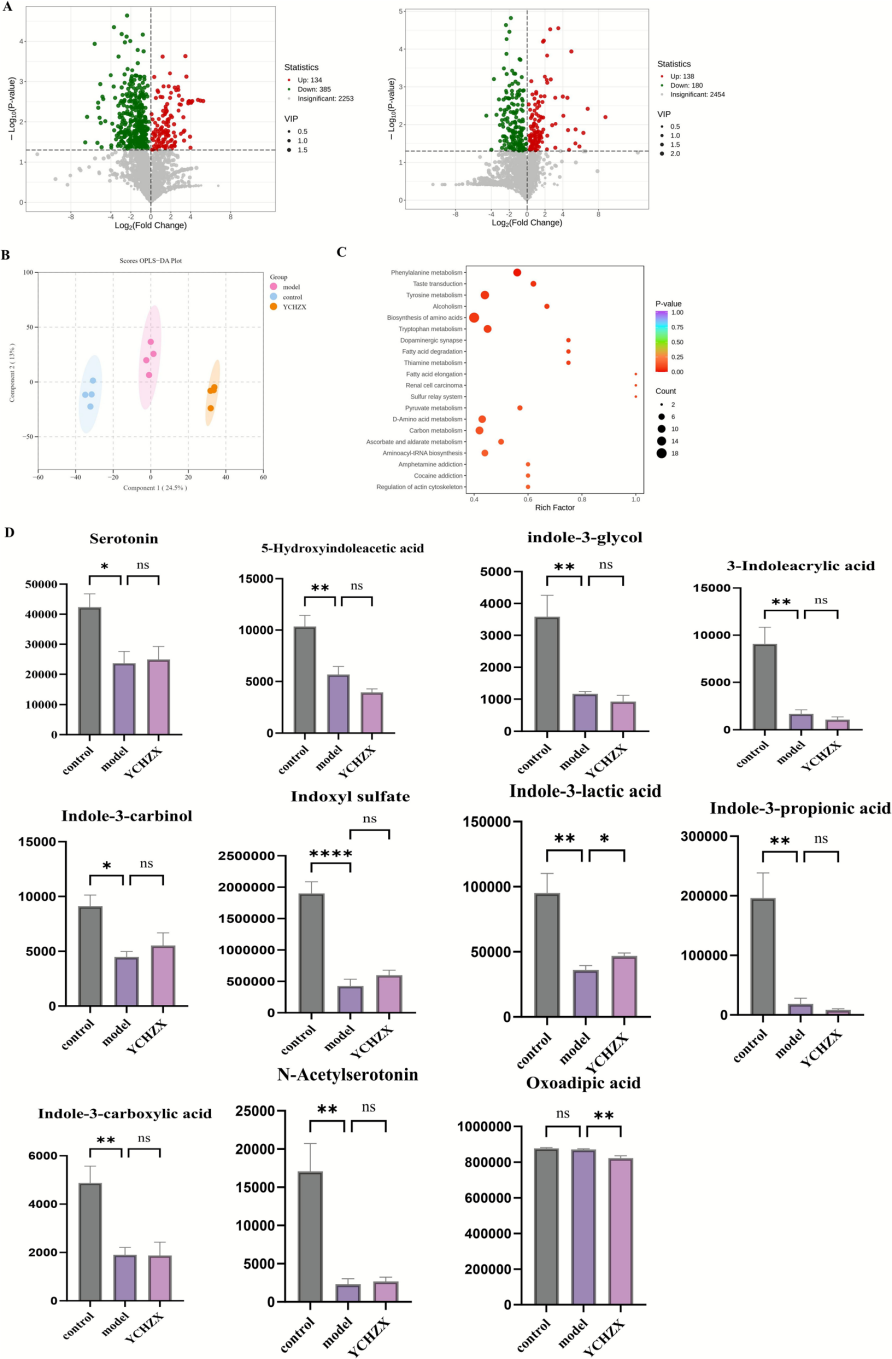
YCHZX modulates tryptophan metabolism. **A** Differential metabolites detected in positive and negative ion modes; **B** OPLS-DA score plots; **C** KEGG pathway analysis of differentially expressed metabolites; **D** Levels of representative differential metabolites (n = 4). Data are presented as mean ± SE. *P < 0.05, **P < 0.01, ***P < 0.001, ****P < 0.0001. YCHZX: Yinchenhao Tang plus Zexie Tang

Untargeted serum metabolomics identified profound metabolic alterations in the model group, with 134 metabolites elevated and 385 reduced compared to the control group. YCHZX treatment partially reversed these changes, increasing 138 and decreasing 180 metabolites relative to the model group (Fig. 5A). OPLS-DA showed clear separation between the control, model, and YCHZX groups (Fig. 5B), confirming distinct metabolic phenotypes. Functional prediction of the gut microbiota using PICRUSt2, complemented by KEGG pathway enrichment analysis of the serum metabolomics, identified several metabolic pathways, including tyrosine metabolism, thiamine metabolism, and tryptophan metabolism (Fig. 5C and Supplementary Fig. 2). Based on this integrative analysis and supporting literature, we prioritized tryptophan metabolism for further investigation. Key tryptophan-derived metabolites—including serotonin, indole-3-carbinol (I3C), indole-3-glycol, 3-indoleacrylic acid (3-IDC), 5-hydroxyindoleacetic acid (5-HIAA), indoxyl sulphate (IS), ILA, indole-3-carboxylic acid (3-ICA), and indole-3-propionic acid (IPA)—were significantly lower in the model group than in the control group, YCHZX intervention markedly elevated ILA levels while concurrently reducing oxoadipic acid (Fig. 5D and Supplementary Table 2).

### YCHZX targets butyrate producer-associated IDO1 suppression

**Fig. 6:**
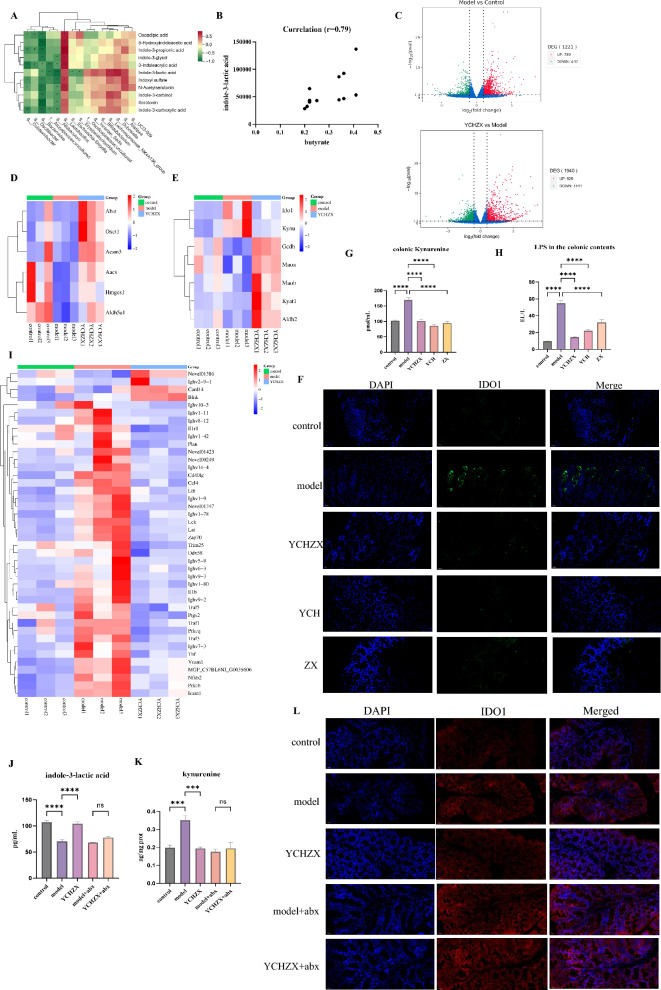
YCHZX targets butyrate producer-associated IDO1 suppression. **A** Integrated analysis of metabolomic and gut microbiota data (n = 12); **B** Correlation between indole-3-lactic acid and butyrate levels (n = 12); **C** Differentially expressed genes; **D** Heatmap of butyrate metabolism-related genes; **E** Heatmap of tryptophan metabolism-related genes; **F** Immunofluorescence staining of IDO1 (green) in colon Sects. (400 ×); **G** Colonic kynurenine levels (n = 6); **H** LPS levels in colonic contents (n = 6); **I** Heatmap of inflammation-related gene expression (n = 3); **J** Serum indole-3-lactic acid levels in PGF mice (n = 5); **K** Colonic kynurenine levels in PGF mice(n = 5); **L** Immunofluorescence staining of IDO1 (red) in colon sections of PGF mice (400 ×). Nuclei were counterstained with DAPI (blue). Data are presented as mean ± SE. *P < 0.05, **P < 0.01, ***P < 0.001, ****P < 0.0001. YCHZX: Yinchenhao Tang plus Zexie Tang; YCH: Yinchenhao Tang; ZX: Zexie Tang; IDO1: indoleamine 2,3-dioxygenase; NASH: nonalcoholic steatohepatitis; PGF: pseudogerm-free

Correlation analysis revealed a positive correlation between the abundance of the butyrate-producing genus *Lachnospiraceae_NK4A136_group* and serum ILA levels, in contrast to the negative correlation observed between the LPS-producing taxon *Escherichia–Shigella* and ILA (Fig. 6A). Supporting these findings, functional prediction via PICRUSt2 indicated significant enrichment of LPS biosynthesis pathways in the model group, whereas YCHZX treatment potently enhanced butanoate metabolism (Supplementary Fig. 2). Given the established role of *Lachnospiraceae_NK4A136_group* as a butyrate producer [35] and the observed positive correlation between colonic butyrate and ILA (Fig. 6B), we hypothesized that butyrate promotes ILA accumulation by suppressing IDO1—the rate-limiting enzyme that diverts tryptophan into the kynurenine pathway. This hypothesis aligns with prior evidence that butyrate alleviates Proteobacteria-driven colonic immune activation and downregulates IDO1 expression in high-fat diet models [36], and that it can directly inhibit IDO1 [37], suggesting a conserved regulatory mechanism likely operative in our NASH context.

To systematically characterize host transcriptional responses, we performed colon transcriptomic profiling. This analysis revealed extensive gene dysregulation in the model group, with 789 genes upregulated and 432 downregulated relative to controls. YCHZX intervention altered the expression of 1,940 genes (829 upregulated and 1,111 downregulated) compared to the model group (Fig. 6C). Notably, YCHZX administration restored butanoate metabolism pathways (Fig. 6D). Critically, YCHZX suppressed IDO1 expression, as evidenced by reduced IDO1 transcription (Fig. 6E), diminished protein expression on immunofluorescence (Fig. 6F), and decreased colonic kynurenine concentrations (Fig. 6G). The induction of IDO1 was closely associated with Proteobacteria-mediated inflammation, which was further supported by elevated colonic LPS levels (Fig. 6H) and the activation of inflammatory gene programs (Fig. 6I). Crucially, antibiotic-mediated microbiota ablation confirmed the gut microbiota-dependence of these effects: YCHZX increased serum ILA and suppressed IDO1 expression and colonic kynurenine in microbiota-intact mice, whereas these benefits were diminished upon microbiota depletion (Fig. 6J–L). Complementing these in vivo observations, sodium butyrate treatment reduced IDO1 protein expression and kynurenine levels in LPS-stimulated Caco-2 cells (Supplementary Fig. 3A). Collectively, these findings support a model in which butyrate, derived from YCHZX-modulated microbiota, contributes to IDO1 suppression through a dual mechanism: indirectly by mitigating *Escherichia–Shigella*-associated inflammation, and directly by inhibiting IDO1 in host intestinal cells.

### YCHZX enhances AhR-mediated barrier function via gut bacteria-dependent ILA signaling

**Fig. 7:**
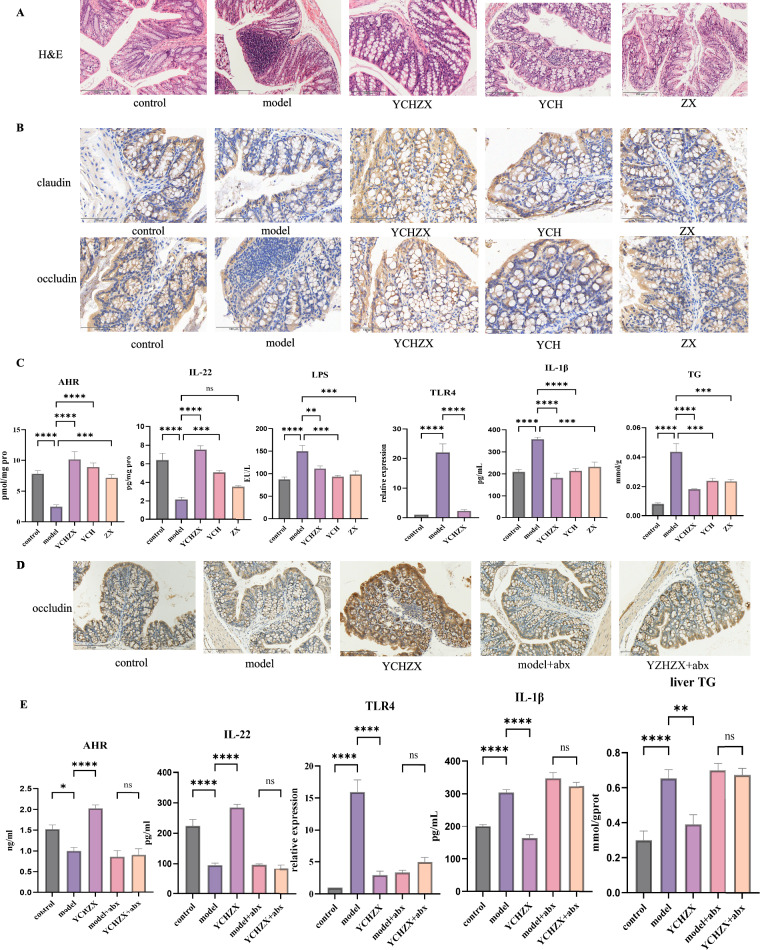
YCHZX enhances AhR-mediated barrier function via gut microbiota-dependent ILA signaling. **A** H&E staining of colon tissues (200 ×); **B** Immunohistochemistry of claudin and occludin in colon Sects. (200 ×); **C** Levels of AhR and IL-22 in colon, serum LPS, hepatic TLR4 mRNA, IL-1β, and TG (n = 5–6); **D** Immunohistochemistry of occludin in colon sections of PGF mice (200 ×); **E** Levels of AhR and IL-22 in colon, hepatic TLR4 mRNA, IL-1β, and TG in PGF mice (n = 5). Data are presented as mean ± SE. *P < 0.05, **P < 0.01, ***P < 0.001, ****P < 0.0001. YCHZX: Yinchenhao Tang plus Zexie Tang; YCH: Yinchenhao Tang; ZX: Zexie Tang; AhR: aryl hydrocarbon receptor; IL-22: interleukin-22; TLR4: Toll-like receptor 4; TG: triglyceride; IL-1β: interleukin-1β; NASH: nonalcoholic steatohepatitis; PGF: pseudogerm-free

Given that ILA has been reported as an endogenous ligand of the AhR in previous studies [38], and that AhR activation is known to promote gut barrier integrity and reduce LPS translocation, we evaluated whether YCHZX modulates this ILA-AhR pathway. Histological analysis indicated severe colonic damage in the model group, characterized by loss of goblet cells, epithelial disruption, and inflammatory infiltration. These pathological changes were substantially attenuated by YCHZX intervention (Fig. 7A). Immunohistochemistry further confirmed that YCHZX restored the expression of the tight junction proteins claudin and occludin (Fig. 7B). Consistent with the hypothesized ILA-AhR axis, YCHZX increased colonic levels of AhR and IL-22, while reducing serum LPS (Fig. 7C). Downstream hepatic analysis revealed suppressed TLR4 mRNA expression along with decreased TG and IL-1β levels (Fig. 7C). Importantly, antibiotic-induced microbiota depletion diminished the beneficial effects of YCHZX: the upregulation of occludin, activation of AhR pathway, and inhibition of the TLR4 pathway were all attenuated in microbiota-depleted mice (Fig. 7D, E). We treated LPS-induced Caco-2 cells with ILA in the presence or absence of the AhR antagonist CH223191. First, concentration-dependent effects on cell viability were evaluated for the ILA and the AhR antagonist CH223191. Since cell viability remained above 80% at 0.5 mmol/L ILA and exceeded 90% at 10 μmol/L CH-223191 (Supplementary Fig. 3B), and in accordance with previous studies [30–32], these concentrations (0.5 mmol/L for ILA and 10 μmol/L for CH-223191) were set as the working concentrations for subsequent experiments. The AhR was significantly activated by ILA and inhibited by CH223191, indicated by the upregulation of Cyp1a1 (Supplementary Fig. 3C). ILA significantly enhanced the expression of occludin, an effect that was attenuated upon inhibition of AhR, demonstrating that the effects of ILA are AhR-dependent (Supplementary Fig. 3C). These results suggest that YCHZX enhances intestinal barrier function and suppresses hepatic inflammation through a gut bacteria-dependent ILA-AhR axis.

## Discussion

NASH, a progressive subtype of NAFLD, poses a substantial and growing global health burden [39]. The multifactorial pathogenesis of NASH—encompassing gut microbiota dysbiosis, metabolic reprogramming, and gut–liver axis dysregulation—demands interventions capable of systemically targeting these interconnected networks. TCM formulations, characterized by their multi-component and multi-targeted nature, have shown promise in treating metabolic diseases such as NASH. However, the absence of systematic mechanistic insights has impeded clinical translation. In this study, we applied an integrated multi-omics approach—including 16S rRNA sequencing, serum metabolomics, and colon transcriptomics—together with functional validation to delineate the therapeutic mechanism of YCHZX in a HFHFC diet-induced NASH mouse model, which recapitulates key pathological features of human NASH [26]. Our results define a previously unreported, gut microbiota-dependent regulatory axis wherein YCHZX alleviates NASH by enriching specific butyrate-producing bacteria, suppressing colonic IDO1, and reprogramming tryptophan metabolism toward ILA production.

A central finding is that YCHZX orchestrates a unique gut microbiota structure in NASH mice, notably enriching butyrate-producing genera such as *Lachnospiraceae_NK4A136_group*—a major source of short-chain fatty acids that strengthen intestinal barrier function and attenuate pro-inflammatory responses [40]. This mechanism distinguishes YCHZX from other TCM formulations. For instance, ZX primarily enhances *Akkermansia muciniphila* abundance and modulates bile acid metabolism, exerting therapeutic effects via G protein-coupled bile acid receptor 1(GPBAR1)-mediated signaling in adipose tissue [24]. Similarly, YCH exerts therapeutic effects via decreasing Bacteroides_vulgatus abundance and Palmitoleic acid[41]. Critically, our study reveals that YCHZX not only restores *Lachnospiraceae_NK4A136_group*—a genus commonly depleted in human and experimental NASH [42, 43]—but also functionally couples this enrichment to the suppression of colonic IDO1, thereby redirecting tryptophan metabolism from the kynurenine pathway toward ILA generation. The entire pathway proved microbiota-dependent, as evidenced by its diminishment following antibiotic-induced microbiota depletion.

Integrative multi-omics analyses further elucidated the mechanistic connections between YCHZX-driven enrichment of butyrate producers and NASH improvement. Serum metabolomics-based KEGG pathway analysis and functional prediction (PICRUSt2) of the gut microbiota highlighted tryptophan metabolism as a key pathway affected by YCHZX, marked by specific restoration of ILA. Correlation analyses revealed positive associations among *Lachnospiraceae_NK4A136_ group* abundance, colonic butyrate levels, and serum ILA. Transcriptomics and subsequent experimental validation confirmed that YCHZX significantly suppresses colonic IDO1—a pivotal enzyme that diverts tryptophan toward the pro-inflammatory kynurenine pathway, thereby limiting production of beneficial indole metabolites such as ILA [44]. These findings were supported by in vitro experiments showing that sodium butyrate reduces IDO1 protein expression and kynurenine levels in LPS-stimulated Caco-2 cells. Our data further supported this axis: antibiotic-mediated microbiota ablation diminished YCHZX-induced increases in IDO1 suppression, and ILA restoration. Although previous studies have separately implicated butyrate in NASH improvement [45] or ILA in gut barrier enhancement via aryl AhR activation [38]. This finding provides a novel "butyrate–IDO1–ILA" regulatory cascade in NASH. Butyrate, derived from YCHZX-remodeled microbiota, may serve as a physiological negative regulator, corroborating prior evidence from other models [36].

We further investigated the downstream consequences of ILA restoration on gut–liver axis homeostasis. ILA serves as an endogenous ligand of AhR [38], a transcription factor vital to gut barrier maintenance and immune regulation. YCHZX elevated colonic AhR levels, enhanced expression of occludin, and reduced serum LPS translocation—effects indicative of AhR activation and barrier repair. In the liver, YCHZX correlated with suppression of LPS/TLR4 signaling, hepatic triglyceride accumulation, and IL-1β production—central drivers of NASH progression [7]. These benefits were diminished in microbiota-depleted mice. Using LPS-induced Caco-2 cells, ILA activated the AhR pathway (evidenced by Cyp1a1 upregulation) and strengthened barrier integrity, effects abolished by the AhR antagonist CH223191. These results suggest that YCHZX enhances intestinal barrier function and alleviates hepatic inflammation through a gut microbiota–dependent ILA–AhR pathway.

Despite the mechanistic insights provided by this study, several limitations warrant consideration and highlight avenues for future investigation. Although our in vitro findings demonstrate that sodium butyrate suppresses IDO1 and that ILA-dependent barrier enhancement requires AhR activation, the functional relevance of these pathways within the complex in vivo gut microenvironment remains to be fully established. Future studies employing NASH models treated with butyrate, combined with intestinal epithelium-specific AhR or IDO1 knockout mice, would be crucial to conclusively define the contribution of this host signaling axis to the effects of YCHZX-remodeled microbiota. Second, the specific phytochemicals in YCHZX responsible for enriching butyrate-producing bacteria have yet to be definitively identified. Although geniposide and alisol B emerge as lead candidates—supported by computational docking (Supplementary Fig. 4) and literature evidence[46, 47] —their causal role requires direct experimental confirmation. An approach would be to combine in vitro gastrointestinal digestion of YCHZX with anaerobic fecal fermentation assays, comparing the ability of pre- and post-digestion samples to promote the growth of key taxa such as the *Lachnospiraceae_NK4A136_group*. Subsequent validation in gnotobiotic models would be essential to pinpoint the true active constituents. Third, the clinical translatability of our findings remains to be established. Our conclusions are based on a murine NASH model, which does not fully capture the heterogeneity of human disease, including individual variations in gut microbiota and common comorbidities. Profiling microbial, metabolic, and transcriptional changes in NASH patients treated with YCHZX will be a critical step toward verifying the clinical relevance of the proposed mechanism.

**Fig. 8:**
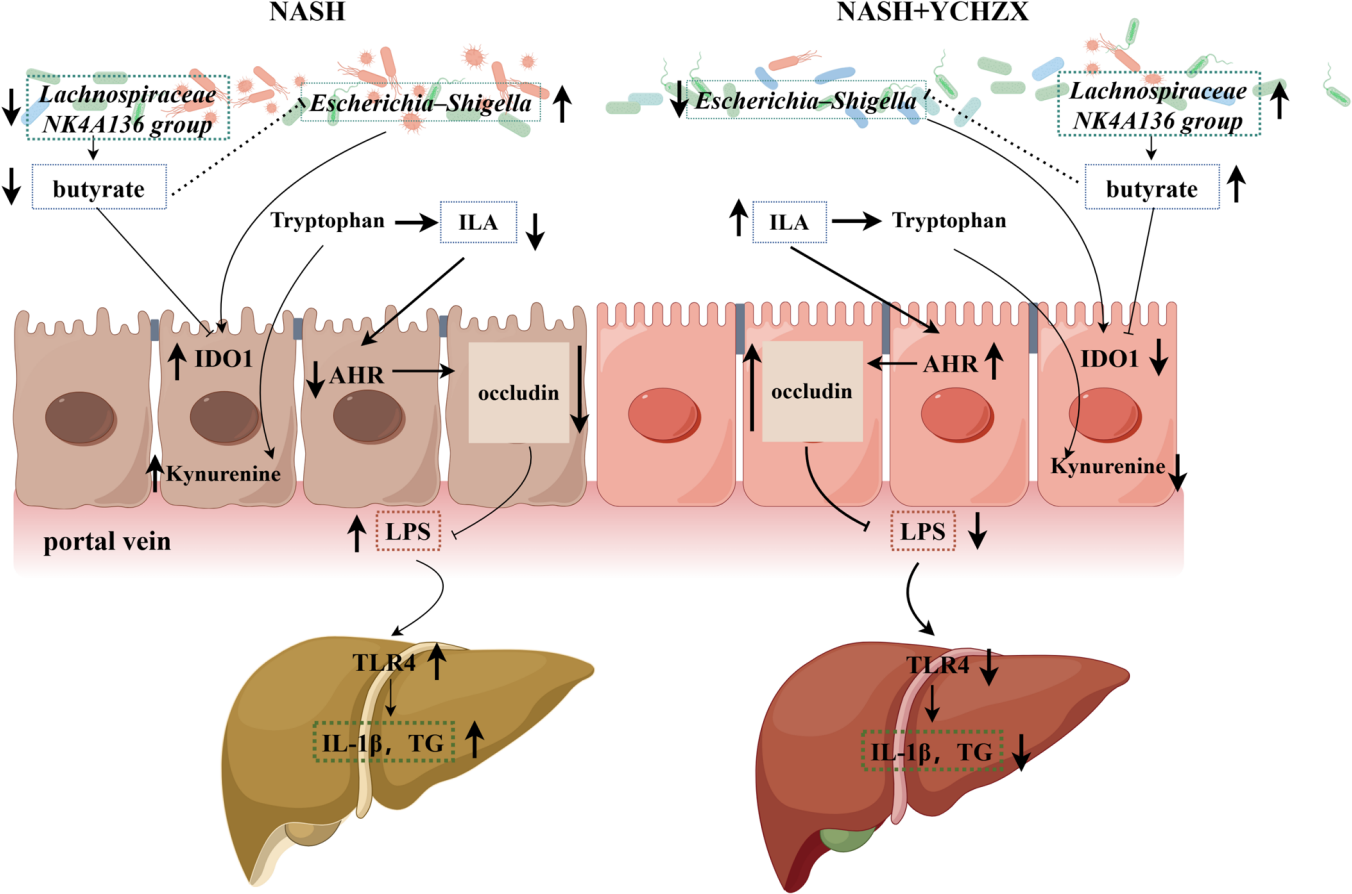
Proposed mechanism: YCHZX alleviates NASH via a gut microbiota-dependent mechanism: enriching butyrate-producing bacteria is associated with suppression of colonic IDO1, which reprograms tryptophan metabolism toward ILA, and subsequently activates the AhR to restore gut barrier function and inhibit hepatic inflammation. YCHZX: Yinchenhao Tang plus Zexie Tang; YCH: Yinchenhao Tang; ZX: Zexie Tang; IDO1: indoleamine 2,3-dioxygenase; ILA: indole-3-lactic acid; AhR: aryl hydrocarbon receptor; TLR4: Toll-like receptor 4; TG: triglyceride; IL-1β: interleukin-1β; NASH: nonalcoholic steatohepatitis

In summary, our integrated multi-omics study demonstrates that YCHZX ameliorates NASH in a gut microbiota-dependent manner. The therapeutic effect is linked to a mechanistic model involving: (1) enrichment of butyrate-producing genera (e.g., *Lachnospiraceae_NK4A136_group*), correlating with increased colonic butyrate; (2) suppression of colonic IDO1, which is associated with a metabolic shift in tryptophan metabolism from the kynurenine pathway toward ILA production; (3) activation of the AhR pathway, which contributes to the restoration of gut barrier integrity and reduced LPS translocation; and (4) subsequent inhibition of hepatic LPS/TLR4 signaling and inflammation (Fig. 8). This work provides evidence for a “butyrate-IDO1-ILA” axis as a potential target of TCM in NASH, highlighting the modulation of gut microbiota-host interactions as a promising therapeutic strategy.

## Supplementary Information

Supplementary Material 1.

Supplementary Material 2.

Supplementary Material 3.

Supplementary Material 4.

Supplementary Material 5. Chemical constituents of YCHZX.

Supplementary Material 6. Detailed characterization data for the differential metabolites.

## Data Availability

The data used to support the findings of this study are available from the corresponding author upon request.

## Abbreviations

3-ICA: Indole-3-carboxylic acid
3-IDC: 3-Indoleacrylic acid
5-HIAA: 5-Hydroxyindoleacetic acid
AhR: Aryl hydrocarbon receptor
ALT: Alanine aminotransferase
ASVs: Amplicon sequence variants
AST: Aspartate aminotransferase
BSA: Bovine serum albumin
Cyp1A1: Cytochrome P450 Family 1 Subfamily A Member 1
ELISA: Enzyme-linked immunosorbent assay
GPBAR1: G protein-coupled bile acid receptor 1
HCC: Hepatocellular carcinoma
HDL-C: High-density lipoprotein cholesterol
H&E: Hematoxylin and eosin
HFHFC: High-fat/high-fructose/high-cholesterol
Hprt1: Hypoxanthine phosphoribosyltransferase 1
IDO1: Indoleamine 2,3-dioxygenase
LEfSe: Linear discriminant analysis effect size
ILA: Indole-3-lactic acid
IL-22: Interleukin-22
IL-1β: Interleukin-1 beta
I3C: Indole-3-carbinol
IPA: Indole-3-propionic acid
IS: Indoxyl sulfate
LDL-C: Low-density lipoprotein cholesterol
LPS: Lipopolysaccharide
NAFLD: Nonalcoholic fatty liver disease
NAFL: Nonalcoholic fatty liver
NASH: Nonalcoholic steatohepatitis
OCT: Optimal cutting temperature compound
OPLS-DA: Orthogonal partial least squares-discriminant analysis
PBS: Phosphate-buffered saline
PGF: Pseudogerm-free
RT–qPCR: Reverse transcription quantitative polymerase chain reaction
SCFAs: Short-chain fatty acids
TCM: Traditional Chinese medicine
TC: Total cholesterol
TG: Triglyceride
TLR4: Toll-like receptor 4
TPH1: Tryptophan hydroxylase 1
UHPLC-Q-Orbitrap HRMS: Ultrahigh-performance liquid chromatography–Q-Orbitrap high-resolution mass spectrometry
YCH: Yinchenhao Tang
YCHZX: Yinchenhao Tang plus Zexie Tang
ZX: Zexie Tang
