# Supplementary material for: Multi-omics reveals an association of the gut butyrate-IDO1-tryptophan axis with Yinchenhaotang plus Zexietang-ameliorated NASH in a microbiota-dependent manner
Source: Chin Med. 2026 Jan 21;21:44. doi: 10.1186/s13020-025-01304-w (PMC12821316; doi:10.1186/s13020-025-01304-w)
Supplement: Supplementary file 1 — Supplementary Material 1. [file 13020_2025_1304_MOESM1_ESM.pdf]

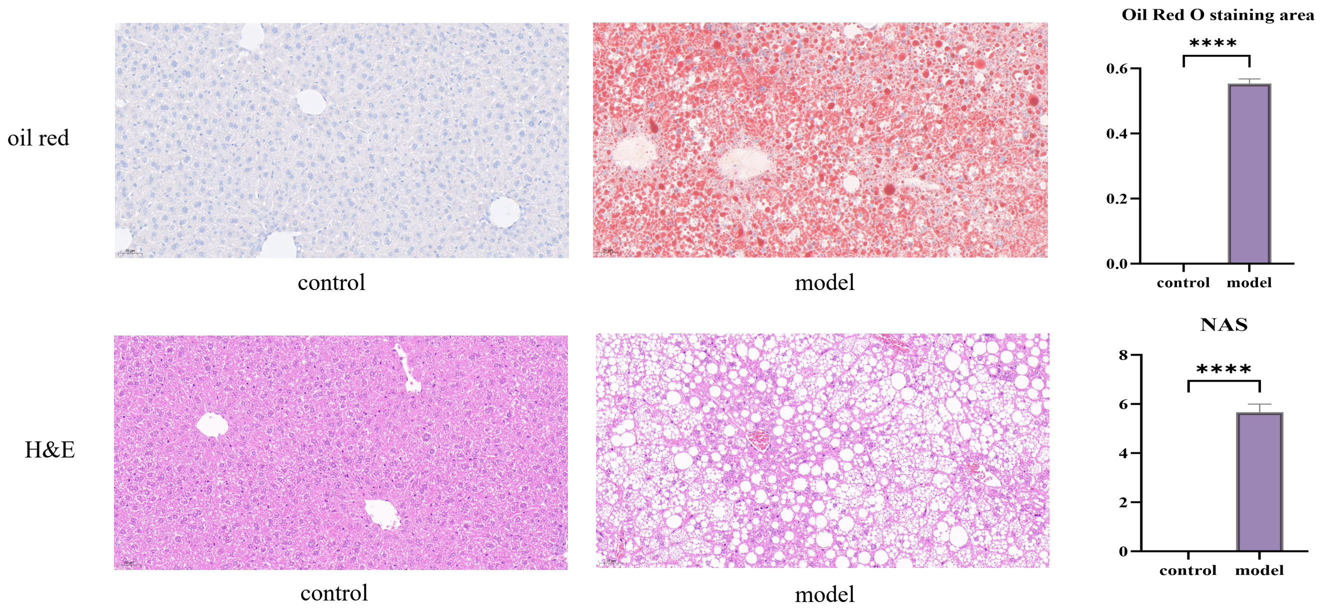

Supplementary figure 1 Oil Red O and H&E staining of liver sections (200×)
Data are presented as mean ± SE(n=3). *P < 0.05, **P < 0.01, ***P < 0.001,****P < 0.0001.
