# Supplementary material for: Multi-omics reveals an association of the gut butyrate-IDO1-tryptophan axis with Yinchenhaotang plus Zexietang-ameliorated NASH in a microbiota-dependent manner
Source: Chin Med. 2026 Jan 21;21:44. doi: 10.1186/s13020-025-01304-w (PMC12821316; doi:10.1186/s13020-025-01304-w)
Supplement: Supplementary file 3 — Supplementary Material 3. [file 13020_2025_1304_MOESM3_ESM.pdf]

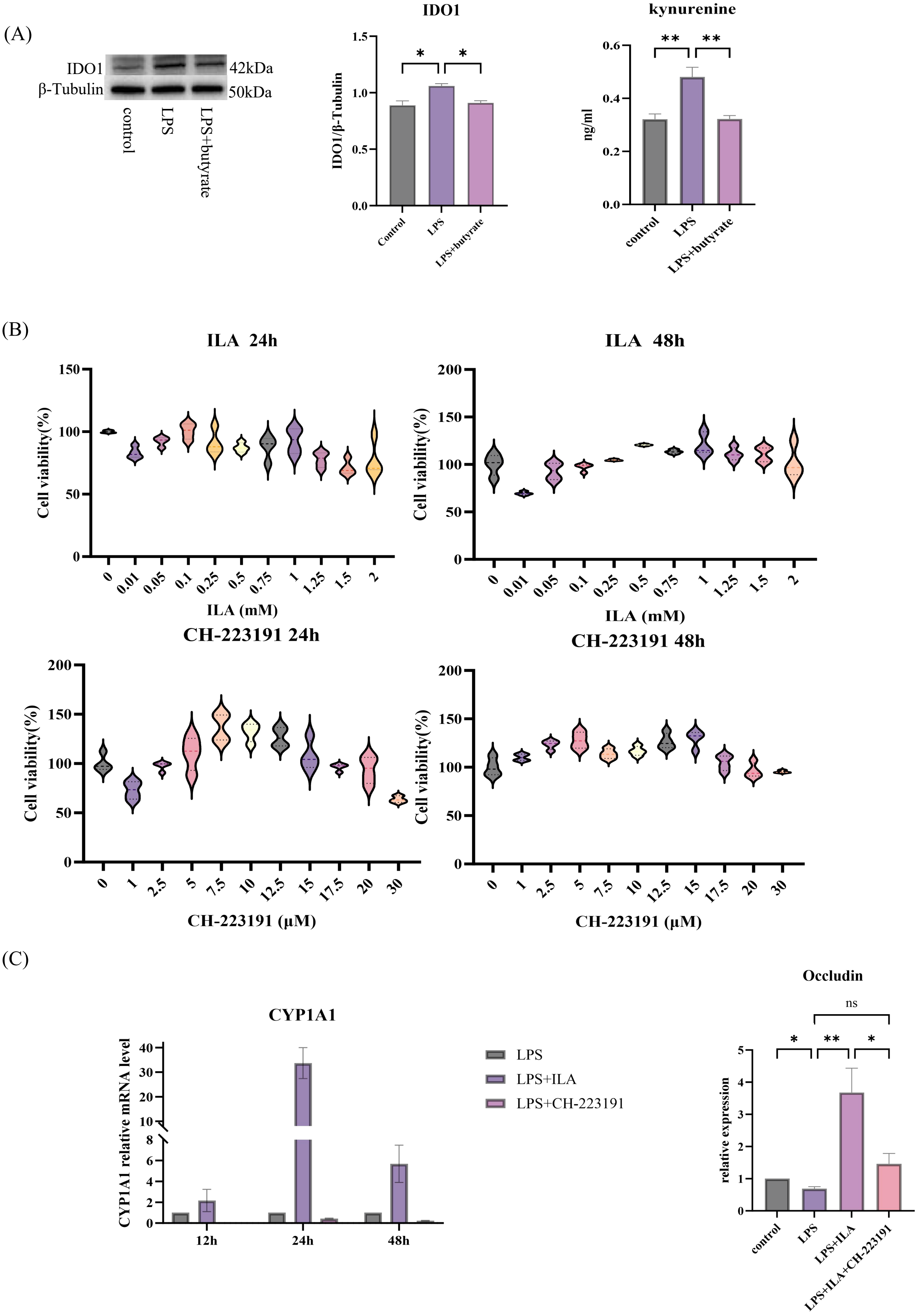

Supplementary figure 3 Results in LPS-stimulated Caco-2 cells.(A) Western blot of IDO1 (n = 3) and kynurenine levels (n = 4) ; (B) Determination of treatment concentrations (n = 4);(C) RT–qPCR analysis of Cyp1A1 and occludin mRNA expression (n = 3). Data are presented as mean ± SE. *P < 0.05, **P < 0.01, ***P < 0.001.
