# Supplementary material for: Multi-omics reveals an association of the gut butyrate-IDO1-tryptophan axis with Yinchenhaotang plus Zexietang-ameliorated NASH in a microbiota-dependent manner
Source: Chin Med. 2026 Jan 21;21:44. doi: 10.1186/s13020-025-01304-w (PMC12821316; doi:10.1186/s13020-025-01304-w)
Supplement: Supplementary file 4 — Supplementary Material 4. [file 13020_2025_1304_MOESM4_ESM.pdf]

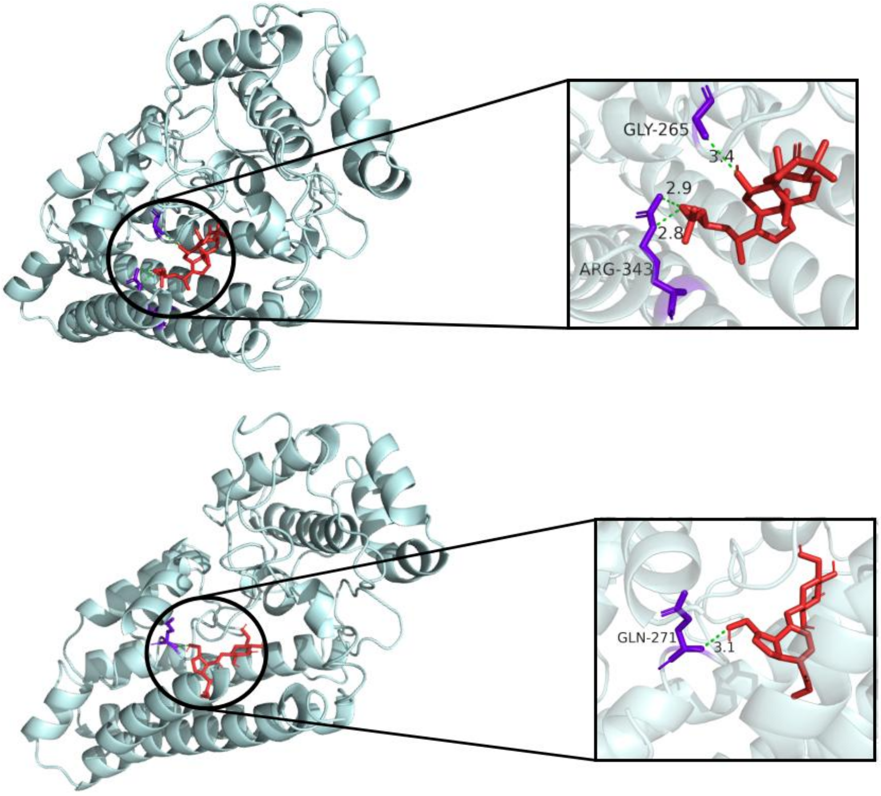

Supplementary figure 4 Predicted binding modes of IDO1 with (A) Alisol B (binding energies of -10.0 kcal/mol) (B) Geniposide (binding energies of -8.2 kcal/mol)
